# Recruitment of Hard-to-Reach Older Adults in Technology-Based Mental Health Services Studies: Lessons Learned

**DOI:** 10.1093/geroni/igaa057.1328

**Published:** 2020-12-16

**Authors:** Patrick Ho Lam Lai, Xiaoling Xiang, Yihang Sun, Joseph Himle

**Affiliations:** 1 University of Michigan-Ann Arbor, Ypsilanti, Michigan, United States; 2 University of Michigan, Ann Arbor, Michigan, United States

## Abstract

Homebound older adults are a hard-to-reach population with a high burden of depression and face substantial access barriers to mental health treatments. Internet-based psychotherapy is a promising strategy to address this persistent treatment gap, but older adults are severely underrepresented in internet-based psychotherapy trials. One challenge to advance this area of research and practice is the recruitment and retention of homebound older adults in clinical trials. Previous research has discussed the challenges of recruiting older adults in behavioral interventions and offered useful recommendations. However, recruiting homebound older adults, who face substantial mobility barriers, poses additional challenges not fully addressed in the literature. The expectation of using technology adds another layer of difficulty. In this presentation, we will discuss our group’s experiences working with community partners to recruit and retain homebound older adults for a study on technology-based mental health treatment. We partnered with home care agencies, senior apartment buildings, and Meals-on-Wheels and experimented with a few different ways to recruit study participants. Issues related to accessibility, trust, and stigma emerged as important considerations when designing recruitment strategies and materials. The discussion will be an integration of our experiences and a review of previous literature on the challenges and recommendations for recruiting older adults in mental health services research.

