# Erratum to: Recruitment of Hard-to-Reach Older Adults in Technology-Based Mental Health Services Studies: Lessons Learned

**DOI:** 10.1093/geroni/igab006

**Published:** 2021-04-20

**Authors:** Patrick Ho Lam Lai, Xiaoling Xiang, Yihang Sun, Joseph Himle

**Affiliations:** 1 University of Michigan-Ann Arbor, Ypsilanti, Michigan, United States; 2 University of Michigan, Ann Arbor, Michigan, United States

In the published abstract, “Recruitment of Hard-to-Reach Older Adults in Technology-Based Mental Health Services Studies: Lessons Learned,” doi: 10.1093/geroni/igaa057.1328” the first author’s name was erroneously listed again as the last author: Patrick Ho Lam Lai, Xiaoling Xiang, Yihang Sun, Joseph Himle, Ho Lam Lai.

The author list has since been corrected to: Patrick Ho Lam Lai, Xiaoling Xiang, Yihang Sun, Joseph Himle.

